# The Physicochemical Parameters, Phenolic Content, and Antioxidant Activity of Honey from Stingless Bees and *Apis mellifera*: A Systematic Review and Meta-Analysis

**DOI:** 10.3390/antiox13121539

**Published:** 2024-12-16

**Authors:** Ana Karen Zaldivar-Ortega, Antonio de Jesús Cenobio-Galindo, Nuria Morfin, Gabriel Aguirre-Álvarez, Rafael G. Campos-Montiel, Nuria Esturau-Escofet, Angel Garduño-García, Juan Carlos Angeles-Hernandez

**Affiliations:** 1 Instituto de Ciencias Agropecuarias, Universidad Autónoma del Estado de Hidalgo, Avenida Universidad Km. 1 s/n Exhacienda Aquetzalpa, Tulancingo 43600, Mexico; ana_saldivar@uaeh.edu.mx (A.K.Z.-O.); antonio_cenobio@uaeh.edu.mx (A.d.J.C.-G.); aguirre@uaeh.edu.mx (G.A.-Á.); rcampos@uaeh.edu.mx (R.G.C.-M.); 2 Michael Smith Laboratories, Department of Biochemistry & Molecular Biology, The University of British Columbia, Vancouver, BC V6T1Z4, Canada; nuriamorfin@ttp-bchpa.ca; 3 Instituto de Química, Universidad Nacional Autónoma de México, Ciudad de México 04510, Mexico; nesturau@iquimica.unam.mx; 4 Departamento de Ingeniería Mecánica Agrícola, Universidad Autónoma Chapingo, Carretera México-Texcoco, Km 38.5, Texcoco 56230, Mexico; 5 Departamento de Medicina y Zootecnia de Rumiantes, Facultad de Medicina Veterinaria y Zootecnia, Universidad Nacional Autónoma de México, Ciudad de México 04510, Mexico

**Keywords:** honey, *Apis mellifera*, stingless bees, bioactive compounds, antioxidant activity, physicochemical composition

## Abstract

The most common bee species used for honey production is *Apis mellifera* (*A. mellifera*), followed by stingless bees. This study included scientific articles using the PRISMA approach. A random effect model was implemented and the effect size (ES) was calculated and reported as the standardized mean difference (SMD) and raw mean difference (RMD). The mean phenolic content in *A. mellifera* honey was 61.21 ± 28.3 mg GAE/100 g and stingless bee honey +33.69 mg GAE/100 g; *p* = 0.01. The antioxidant activity, discovered by the Ferric Reducing Antioxidant Power (FRAP) method, showed a mean of 97.34 ± 7.84 μmol Fe(II)/100 g in *A. mellifera* and stingless bee honey +63.39 μmol Fe(II)/100 g; *p* = 0.009. The physicochemical properties showed significant differences in moisture (*A. mellifera* honey 19.54 ± 3.65%; stingless bee honey +8.02%; *p* = 0.0001), hydroxymethylfurfural (HMF) (*A. mellifera* honey 20.14 ± 16.27 mg/kg; stingless bee honey −11.25 mg/kg; *p* = 0.001), and free acidity (*A. mellifera* honey 31.32 ± 16.67 meq/kg; stingless bee honey +34.76 meq/kg; *p* = 0.01). The variability in the trials was explained by the heterogeneity, and a meta-regression analysis incorporated four covariates: (1) stingless bee species; (2) floral source; (3) country, and (4) latitude. This study highlights the importance of conducting further studies on stingless bee honey.

## 1. Introduction

The most common bee species used for honey production and other bee products (i.e., pollen, beeswax, propolis, and royal jelly) is the species *Apis mellifera* L., followed by stingless bees [1]. Both bees are classified in the family *Apidae* and the subfamily *Apinae*, but the most popular European honey bee is classified in the tribe *Apini*, genus *Apis*, species *mellifera*, while stingless bees are classified within the tribe *Meliponini* [2,3], which has different genera including *Melipona*, *Scaptotrigona*, and *Trigona* [4]. There are more than 500 species of stingless bees found in tropical and subtropical regions, with most of these species found in Latin America, Africa, Asia, and Australia [5,6].

The bee genera and species have been shown to have a significant impact on both the content of phenolic content and physicochemical parameters of honey [7], but this has not been extensively studied. The composition of honey produced by *Apis mellifera* has been studied and its quality parameters have been established. However, this is not the case for honey produced by stingless bees [5,8]. In fact, the presence of honey from stingless bees on the world market is limited compared to honey from *Apis mellifera*, partly as a result of the lack of legislation on the product and information on its nutritional value [2]. Honey standards have been modified to align with the botanical origin, but not according to different species of bees. Thus, it is important that these factors are considered and that quality standards are established for stingless bee honey [9].

The commercial market for *Apis mellifera* honey and stingless bee honey is made up of different types of consumers. For example, between 80 and 90% of the total honey harvested from stingless bees is sold by producers to local buyers and middlemen in the southeast region of Mexico [10], whereas 90% of the honey from *Apis mellifera* produced in the same region is exported to European countries [11]. Additionally, stingless bee honey is commercialized in traditional free markets at significantly higher prices compared to *Apis mellifera* honey [12]. For instance, in 2016, stingless bee honey prices were three times higher compared to conventional honey from *Apis mellifera* [10], which might seem advantageous for meliponiculturists (beekeepers specializing in the management of stingless bees). The honey from stingless bees is widely used in various industries, including food, pharmaceuticals, and cosmetics [13]. On the other hand, stingless bees also play an important ecological role in ecosystem conservation [6,14]. They are the main pollinators of wild and cultivated tropical plants, such as orchids, macadamia nuts, mangoes, and coffee [15,16]. Culturally, stingless bees are important because of the hive products obtained from them, such as honey, pollen, and cerumen, which have been used for medicinal purposes by the indigenous communities in many parts of the world due to their nutritional and therapeutic properties attributed to the presence of phenolic compounds [17,18,19].

Nutritionally, honey is mainly composed of carbohydrates and water, but other important components include proteins, organic acids, enzymes, minerals, amino acids, vitamins, and phenolic compounds [20,21]. Regarding phenolic content, flavonoids and phenolic acids are the main chemical compounds studied in honey [22]. These compounds confer nutraceutical properties to stingless bee honey [12]. Bees transfer phenolic compounds from nectar, pollen or propolis to honey [23]. Phenolic compounds are secondary metabolites synthesized by plants to counteract biotic (e.g., pathogenic or competing species) and abiotic (e.g., UV radiation, desiccation, temperature) stressors and help attract bees as pollinators [24]. Additionally, phenolic compounds can influence the organoleptic properties (i.e., color, taste, or flavor) of honey [21], and they have been used as chemical indicators to determine different botanical and geographical origins of honey [25,26]. Several studies have shown higher phenolic content in honey produced by stingless bees (e.g., *Scaptotrigona* spp. and *Melipona* spp.) compared to *Apis mellifera* honey [1,18,27]. Phenolic compounds have been associated with beneficial antimicrobial, anti-inflammatory, and antioxidant activities [4,19,20,28].

The antioxidant activity of honey refers to the potential to reduce oxidative reactions in food systems and human health [29]. Abu et al. [30] reported that stingless bee honey has higher antioxidant activity compared to *Apis mellifera* honey, and the phenolic compounds contribute to the antioxidant capacity. The phenolic profiles of honey depend on the floral sources visited by the bee and consequently the antioxidant capacity [29]. The antioxidant activity in honey has been demonstrated using different tests, for instance, 1,1-diphenyl-2-picrylhydrazyl (DPPH) scavenging assay and Ferric Reducing Antioxidant Power (FRAP) assay [31]. The phenolic compounds, antioxidant activity, and physicochemical composition of honey are influenced by many factors, such as botanical origin, climate, environmental conditions, geographical area, latitude, season, and bee species [8,18,32,33]. The physicochemical parameters of stingless honey differ from those of *Apis mellifera* honey, such as color, higher moisture content, and higher acidity [2,5]. In contrast, the pH, hydroxymethylfurfural, and diastase activity are typically lower in stingless bee honey compared to *Apis mellifera* honey [8,9,14]. Additionally, the species of bee could have an influence on the variety and concentration of bioactive compounds and, consequently, on the antioxidant activity [12]. The aim of this systematic review was to analyze and compare the phenolic content, antioxidant activity, and physicochemical properties of the honey of *Apis mellifera* and stingless bees.

## 2. Materials and Methods

### 2.1. Search Strategy

**Figure 1:**
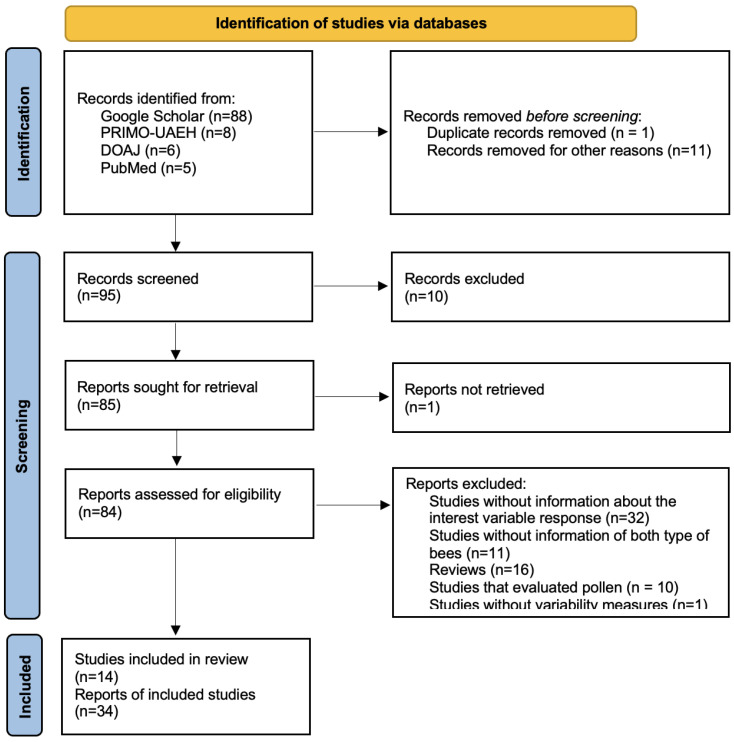
Preferred Reporting Items for Systematic Reviews and Meta-analysis (PRISMA) study flow diagram of the systematic review from the initial search and screening to the final selection of publications to be included in the meta-analysis.

In this study, we conducted an exhaustive and structured search of scientific articles using the predetermined protocol in accordance with the Preferred Reporting Items for Systematic Reviews and Meta-analysis (PRISMA) [34] (Figure 1), and the protocol was registered using the Open Science Framework platform (https://osf.io/s9zt8). The focus of the search was on studies comparing the phenolic compound content, antioxidant activity, and physicochemical composition of honey from *Apis mellifera* versus honey produced by stingless bees. To collect the data, we obtained scientific publications from various databases, including Google Scholar, Primo-UAEH, PubMed, and DOAJ. The article search was performed by three researchers to avoid reviewer bias. A search strategy was developed using the following terms: “honey”, “*Apis mellifera*”, “stingless bees”, “phenols”, “flavonoids”, “antioxidant activity”, and “physicochemical composition” in combination with the Boolean operators (‘and’ or ‘or’). Additionally, some articles were identified from the reference lists of the articles found in the initial search and included in the comprehensive database.

### 2.2. Inclusion Criteria

The parameters of the inclusion criteria were the following:

(a) Studies published in an internationally peer-reviewed scientific journal. Therefore, unpublished manuscripts, conference proceedings, and dissertations were excluded.
(b) Studies that contained information about the physicochemical composition, phenolic content, and antioxidant activity of stingless bees and *Apis mellifera* honey.
(c) Studies that reported the sample size for each group.
(d) Studies that provided least squares means and a measure of data variability such as the coefficient of variation, standard deviation, or standard error.

### 2.3. Database

A total of 14 studies were selected to be included in the meta-analysis procedure, which comprised 34 trials (Figure 1). One researcher extracted the data from the 14 studies that met the inclusion criteria into an Excel spreadsheet (version 2016, Microsoft Corp., Redmond, WA, USA), and two researchers verified the information to identify discrepancies. An Excel spreadsheet was elaborated for each of the following response variables: phenols (mg GAE/100 g), flavonoids (mg QE/100 g), 2,2 diphenyl 1 picrylhydrazyl (DPPH; μmol TE/100 g), Ferric Reducing Antioxidant Power (FRAP; μmol Fe(II)/100 g), moisture (%), hydroxymethylfurfural (HMF; mg/kg), free acidity (meq/kg), color (mm/Pfund), and protein content (g/kg). To explore the sources of between-study variability, the following explanatory variables were selected a priori: the genera of stingless bees (*Heterotrigona*, *Melipona*, *Hypotrigona*, *Plebeia*, *Ptilotrigona*, *Nannotrigona*, *Partamona*, *Scaptotrigona*, and *Trigona*), floral source (monofloral and polyfloral), country (Malaysia, Cuba, Nigeria, Brazil, Peru, and Portugal), and latitude.

### 2.4. Statistical Analysis

The first step of the analytical review was to perform a descriptive analysis using the ‘psych’ package version 2.1.9 [35] in the R statistical computing environment (version 04.2+764, R Core Team, 2024).

#### 2.4.1. Meta-Analysis Procedure

A random effect model was implemented, which assumed that the observed difference among studies was a combination of chance and genuine variation in the intervention effects. The effect size (ES) was calculated and reported as the standardized mean difference (SMD) and raw mean difference (RMD). An SMD allows for the summarization of studies with biologically comparable traits but on different scales. The SMD was calculated according to the method proposed by Hedges [36] and the pooling of analyzed studies was carried out using the inverse variance weighting. The RMD was calculated for response variables reporting outcomes on the same scales, which permits the interpretation of the summary effect under the original measures’ units [37]. All analyses were performed in the R environment for statistical computing (version 04.2+764, R Core Team, 2024) using the package ‘meta’ version 4.13-0 [38].

#### 2.4.2. Assess of Heterogeneity

The current study analyzed honey samples from experiments implemented under different regions, environmental conditions, laboratory methodologies, and experimental designs; hence, the heterogeneity was calculated. Heterogeneity was assessed through the estimation of between-study random effects variance (*t*^2^) and the percentage of variability explained by heterogeneity rather than simple variance (*I*^2^ index), which represents the proportion of between-study variability and is calculated as follows (Equation (1)) [39].

(1) $$I^{2}=\frac{Q-\left(k-1\right)}{Q}\times 100$$

where *Q* is the χ^2^ heterogeneity statistic and *k* is the number of trials. *I*^2^ values of 25%, 50%, and 75% represent small, moderate, and high levels of heterogeneity, respectively.

#### 2.4.3. Meta-Regression

The first step of the meta-regression analysis was to incorporate all explanatory variables in a full model; secondly, multi-predictor models were manually reduced by the backward selection of variables until all predictors were significant (*p* < 0.05). Mixed-effects regression models (meta-regression analysis) were constructed in the “metafor” package. Models were compared and selected by means of information-theoretic criteria using Akaike´s Information Criterion and Bayesian Information Criterion; also, the goodness of fit was evaluated by the coefficient of correlation analog (*R*^2^) for meta-regression which was calculated as follows (Equation (2)):

(2) $$R^{2}=\frac{{\tau^{2}}_{REM-}{\tau^{2}}_{MEM\;}}{{\tau^{2}}_{REM\;}}$$

where ${\tau^{2}}_{REM-}$ is the estimated total heterogeneity based on the random effects model and ${\tau^{2}}_{MEM}$ is the total heterogeneity of the mixed-effects regression model. To evaluate evidence for publication bias, a Begg´s tests were performed [40]. The sensitivity analysis was carried out using a leave-one-out meta-analysis.

## 3. Results

An analytical review and meta-analysis were carried out to assess the effect of the type of bee (*Apis mellifera* vs. stingless bees) on the phenolic content (phenols and flavonoids), antioxidant activity (DPPH and FRAP assays), and physicochemical composition (moisture, HMF, free acidity, color, and protein) of honey. The main causes of article exclusion were studies without results for the response variables of interest (*n* = 34), articles without comparison of both types of bees (*n* = 11), review articles (*n* = 16), studies that evaluated pollen (*n* = 10), and articles without measures of variability (*n* = 1). After applying the inclusion and exclusion criteria, a total of 14 articles published between 2000 and 2022 were included in the meta-analysis (Figure 1). After a sensitivity analysis using the leave-one-out meta-analysis, the pooled result was made stable. No single study had a disproportionate effect on the summary estimate.

### 3.1. Results of Meta-Analysis

#### 3.1.1. Phenols, Flavonoids, and Antioxidant Activity

**Figure 2:**
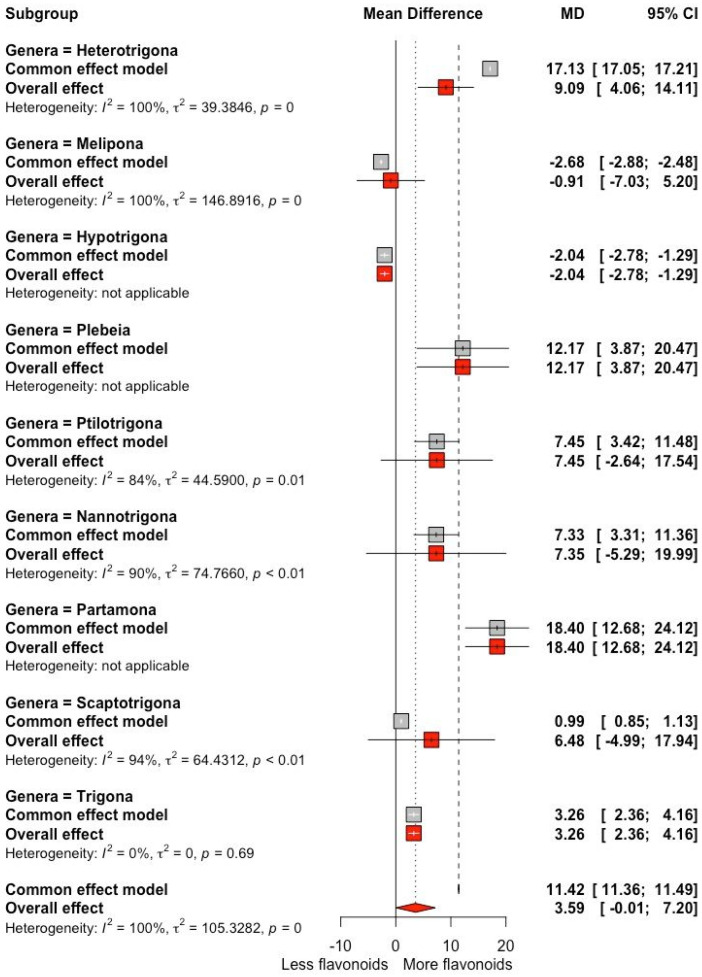
Forest plot of flavonoid content (mg QE/100 g) for trials that included different genera of from stingless bees. The point size reflects the relative weighting of the study to the overall effect size estimate, where a larger point size represents a greater weight in the combined effect size estimated, including the confidence intervals. The diamond represents the overall effect as the raw mean difference (RMD).

The RMD, SMD, heterogeneity, and bias of the response variables are presented in Table 1. The honey produced by stingless bees showed a higher phenolic content compared to *Apis mellifera* honey with an RMD of +33.69 mg GAE/100 g (*p* = 0.01; CI 95% +7.11, +60.27 mg GAE/100 g) and a high heterogeneity (*I*^2^ = 90.4%). The flavonoid content in honey from the different genera of stingless bees was higher (Figure 2) compared to honey from *Apis mellifera* by +3.59 mg QE/100 g (*p* = 0.05; CI 95% −0.01, +7.19 mg QE/100 g), with a high heterogeneity (*I*^2^ = 89.3%) (Table 1).

**Figure 3:**
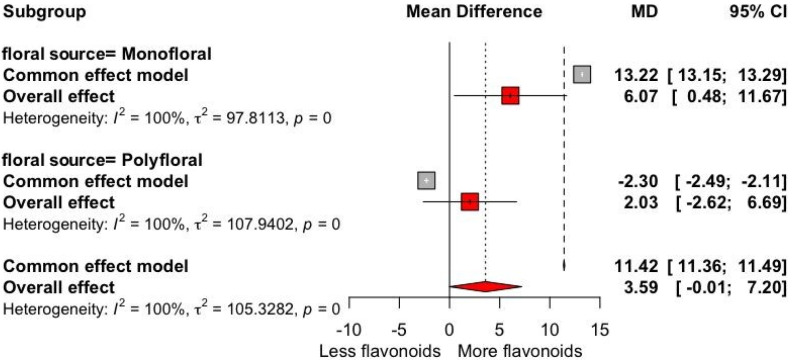
Forest plot of flavonoid content (mg QE/100 g) for floral source trials divided into monofloral and polyfloral origins. The point size reflects the relative weighting of the study to the overall effect size estimate, where a larger point size represents a greater weight in the combined effect size estimated, including the confidence intervals. The diamond represents the overall effect as the raw mean difference (RMD).

Figure 3 shows the mean difference in flavonoid content between the two types of honey of both monofloral and polyfloral origin. The honey of monofloral origin showed a mean difference of 13.22 (95% CI: [13.15, 13.29]), suggesting a higher flavonoid content in this type of honey and a combined effect of 6.07 (95% CI: +0.48, +11.67). Our results depict a significant difference in the antioxidant activity based on the FRAP values among types of bees, with a high antioxidant potential of stingless bee honey (+63.39 μmol Fe(II)/100 g; *p* = 0.009) (Table 1).

**Figure 4:**
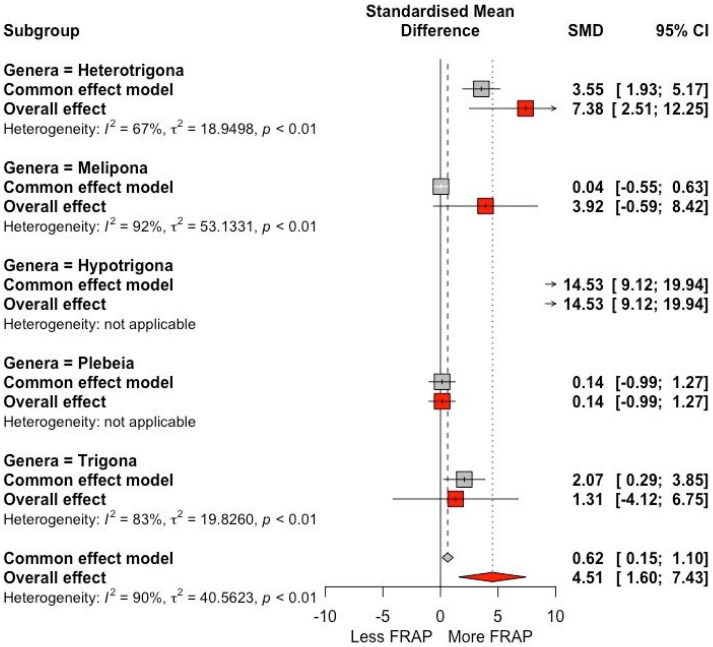
Forest plot antioxidant activity with FRAP (μmol Fe (II)/100 g) for trials that included different genera of stingless bees. The point size reflects the relative weighting of the study to the overall effect size estimate, where a larger point size represents a greater weight and the combined effect size estimated, including the confidence intervals. The diamond represents the overall effect as the raw mean difference (RMD).

The antioxidant activity based on the FRAP value shows an SMD with a significant difference among stingless bee genera (Figure 4). The genus *Heterotrigona* showed an SMD of 3.55 (95% CI: +1.93, +5.17), suggesting a significant increase in antioxidant activity. The overall effect was 7.38 (95% CI: +2.51, +12.25), also indicating a large increase in antioxidant activity (*I*^2^ = 67%.). The genus *Hypotrigona* showed a high effect with an SMD of 14.53 (95% CI: +9.12, +19.94), suggesting an increase in antioxidant activity in this bee species. The heterogeneity of the studies was high, showing that the results varied between studies.

**Figure 5:**
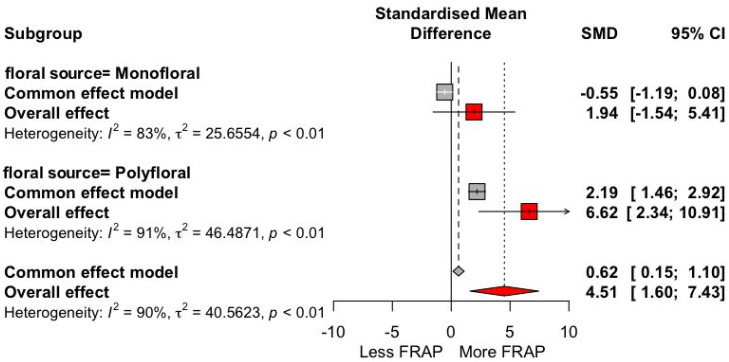
Forest plot of antioxidant activity with FRAP (μmol Fe (II)/100 g) floral source trials, divided into monofloral and polyfloral origin. The point size reflects the relative weighting of the study to the overall effect size estimate, where a larger point size represents a greater weight in the combined effect size estimated, including the confidence intervals. The diamond represents the overall effect as the raw mean difference (RMD).

The antioxidant activity was analyzed according to the floral origin of the honey, divided into monofloral and polyfloral (Figure 5). The results indicated an SMD of 2.19 (95% CI: +1.46, +2.92), with a higher overall effect of 6.62 (95% CI: +2.34, +10.91), showing a statistically significant increase in antioxidant activity in polyfloral honey (*I*^2^ > 90%).

#### 3.1.2. Physicochemical Composition of Honey

The meta-analysis showed significant differences in the physicochemical parameters (Table 1). The moisture (%) was higher in stingless bee honey by +8.02% (*p* = 0.0001; CI 95%: +5.93, +10.11%), with the highest SMD (+10.09; *p* = 0.0001) compared to *Apis mellifera* honey. Additionally, stingless bee honey showed a higher free acidity compared to *Apis mellifera* honey by +34.76 meq/kg (*p* = 0.01; CI 95%: +7.17; +62.34). The color intensity was higher in stingless bee honey compared to *Apis mellifera* (+10.24; CI 95%: +10.22, +30.71), with evidence of significant heterogeneity (*I*^2^ = 95.1%). Conversely, stingless bee honey showed a lower content of HMF (−11.25 meq/100 g; *p* = 0.001; CI 95%: −15.88, −6.62) compared to *Apis mellifera* honey.

### 3.2. Meta-Regression Analysis

A meta-regression analysis was performed to explain the significant heterogeneity among the studies (*I*^2^ > 90%) for the variables analyzed, the physicochemical parameters, phenolic content, flavonoids, and antioxidant activity. Four covariates were identified a priori to explain the variability: (1) stingless bee species; (2) floral source for honey production; (3) country, and (4) latitude. The slopes of the meta-regression and the statistical significance of the covariates, for heterogeneity levels of *I*^2^ > 25%, are shown in Table 2. Various bee genera, the floral source, and country influenced the phenolic content. The results of the meta-regression showed that the genera *Melipona* could produce honey with lower phenolic content (−26.01), (*p* < 0.05). Regarding the country, Cuba (*β* = +69.58; *p* < 0.001) and Peru (*β* = +82.04; *p* < 0.001) were associated with a significant increase in the phenolic content of honey. Latitude also had an effect, as seen in Nigeria (*β* = −1.19; *p* < 0.01), which showed a decrease in the phenolic content.

The flavonoid content was significantly higher in stingless bee honey from Cuba (*β* = +18.07; *p* < 0.001), Nigeria (*β* = +19.87; *p* < 0.001), and Peru (*β* = +15.31; *p* < 0.001) compared to honey from Malaysia, Brazil, and Portugal. In this sense, the country significantly impacted the phenolic content of honey.

Our results showed a positive relationship between the *Hypotrigona* genera and antioxidant activity (*β* = +12.11; *p* < 0.05). The polyfloral source showed a positive association with the high antioxidant activity of honey (*β* = +13.60; *p* < 0.001). The honey from the polyfloral source had a higher moisture percentage (*β* = +14.65; *p* < 0.05) compared to the honey from the monofloral source. Additionally, the moisture was significantly lower in honey from Cuba (*β* = −15.02; *p* < 0.01), Nigeria (*β* = −16.87; *p* < 0.01), and Peru (*β* = −9.89; *p* < 0.05) compared to the rest of the studied locations. Additionally, the hydroxymethylfurfural content in stingless bee honey was lower for honey with a polyfloral botanical origin compared to honey from monofloral sources (*β* = −3.91; *p* < 0.001). Furthermore, the free acidity was lower in honey from the species *Melipona* (*β* = −7.76; *p* < 0.001), *Scaptotrigona* (*β* = −5.09; *p* < 0.05), and *Trigona* (*β* = −45.95; *p* < 0.01) compared to *Heterotrigona*, *Hypotrigona*, *Plebeia*, *Ptilotrigona*, *Nannotrigona*, and *Partamona* bees. The honey that showed the highest free acidity was produced in Brazil (*β* = +6.14; *p* < 0.001) and Cuba (*β* = +2.95; *p* < 0.05), and the honey from Nigeria had the lowest values of free acidity (*β* = −10.85; *p* < 0.001) compared to the rest of the countries. The color intensity was significantly lower in honey from Brazil (*β* = −69.59; *p* < 0.05) and Nigeria (*β* = −30.80; *p* < 0.05). Lastly, our results revealed that the phenolic content (*β* = −1.19; *p* < 0.01), flavonoids *(β* = −0.46; *p* < 0.01), free acidity (*β* = −0.31; *p* < 0.05), and the color intensity (*β* = −2.39 *p* < 0.05) of honey were negatively correlated with latitude.

## 4. Discussion

The current analytical review evaluated the phenolic content (phenols and flavonoids), antioxidant activity (DPPH and FRAP assays), and physicochemical composition of honey (moisture, HMF, free acidity, color, and protein) between *Apis mellifera* and nine genera of stingless bees. The species of honey bee can influence the composition of physicochemical and phenolic content. However, the magnitude of the differences in the honey composition depends mainly on environmental factors (latitude), the floral source (polyfloral and monofloral), and the genera of the producing bee [41]. This investigation highlights the importance of conducting more studies on stingless bee honey.

### 4.1. Phenolic Content and Antioxidant Activity

#### 4.1.1. Phenolic Content

The present study reports differences in the phenolic content between *Apis mellifera* and stingless bee honey. We report on the phenolic and flavonoid content as these are considered to be the primary phenolic compounds present in honey [2,42]. These compounds are particularly important in honey bee products because of their association with anti-inflammatory, antimicrobial, and antioxidant activities, as demonstrated in in vitro assays [43,44,45].

The meta-regression analyses depicted that phenolic and flavonoid content could vary according to the country, floral source, latitude, and the bee type (i.e., *Apis mellifera* and stingless bees). In this sense, the bee type influences honey production due to the different morphological and physiological characteristics among bees that could influence the chemical characteristics of honey. For example, stingless bees are smaller than *Apis mellifera* bees, which allows them to pollinate small-sized flowers and gives them access to different pollen and nectar sources [6,46]. *Apis mellifera* has foraging preferences for specific hues and saturations. In contrast, stingless bees show little preference for color hue or saturation [47]. Foraging choices are influenced by foragers returning to the nest, conveying olfactory and gustatory information about the food source used to nestmates [48]. In addition, stingless bees tend to make shorter flights and prefer low flowering plants for nectar collection [49]. Thus, foraging preferences could be related to the species included in this analysis. Furthermore, the storage conditions of honey in the hive could influence its composition; for example, stingless bees build pots with cerumen to store honey [50,51].

Cerumen is made from the secretions of the bees’ abdominal wax glands, mixed with salivary secretions and plant resins collected by foragers [52]. Similarly, honey bees use beeswax produced by their abdominal glands to build the comb used to store honey [53]. In both cases, the nectar collected by foragers is mixed with the bees’ salivary secretions at the time that trophallaxis occurs, a process that is also useful for reducing the water content of nectar [52]. During the handling of nectar to produce honey, the product is exposed to the pollen collected by foragers; indeed, pollen grains are used to determine the floral origin of honey (melissopalynology) [54]. Additionally, stingless bees and honey bees collect resins to produce propolis, which in both cases is used to seal and sanitize the hive [52,55]. Therefore, the phytochemical compounds found in propolis and pollen could influence the chemical composition of honey. In agreement with Wakhungu et al. [56], *Hypotrigona* bees provide good pollination services within a radius of up to 250 m and can collect pollen from diverse floral sources, which could increase the variability of phenolic compounds in honey collected by this bee. Alvarez-Suarez et al. [18] carried out a study comparing 16 honeys from two different species of bees (*Apis mellifera* and *Melipona beecheii*); the honey samples belonged to municipalities in Cuba, selected based on their melliferous and pollination potential as well as their geographical proximity.

Based on the floral maps developed by the National Centre for Apicultural Research of Cuba, which ensure the similarity of plants that serve as pollen and nectar sources for bees, the results showed a significantly higher content of phenolic and flavonoid compounds in honey from *Melipona bececheii* (stingless bee) compared to *Apis mellifera*. Actually, phenolic compounds have been used as chemical indicators to determine different botanical and geographical origins of honey [25,26] and can influence the organoleptic properties (color, taste, or flavor) of honey [23]. Some studies have reported high levels of phenolic compounds in stingless bee honey, like quercetin, kaempferol, p-coumaric acid, hesperidin, ferulic acid, ellagic acid, trans-cinnamic, rutin, catechin, chrysin, and hesperidin [12,29]. Therefore, it is important to mention that the variation in the phenolic and flavonoid content in honey also depends on several factors such as the botanical source of nectar, season, storage conditions, geographical area, environmental conditions, beekeeping management, foraging area, and bee species [2,49,57]. However, further studies on the preferences of honey bees and stingless bees for floral sources and resins, as well as the influence of other hive products such as propolis and pollen on the chemical composition of honey, should be further investigated.

#### 4.1.2. Antioxidant Activity

This systematic review compares the antioxidant activity of *Apis mellifera* and stingless honey using the DPPH and FRAP techniques. The results indicate a higher antioxidant activity in stingless honey compared to *Apis mellifera* honey, as evidenced by the FRAP values. Several studies have linked the phenolic acids and flavonoids in honey to its antioxidant activity [21,28,44]. This activity is strongly influenced by the molecular structure of the phenolic compounds [58], especially the number and arrangement of the hydroxyl groups and the position of the phenolic rings, which are crucial for their antioxidant activity [59]. The antioxidant activity consists of the mechanism by which the antioxidants scavenge or reduce the formation of free radicals (reactive oxygen species—ROS) in the cell [42,58]. The antioxidants protect cells from oxidative damage caused by free radicals and contribute to the prevention of certain diseases [59], including cardiovascular disease, aging, heart disease, cancer, and inflammation [58]. The antioxidant activity in honey can be estimated using different techniques, such as the DPPH and FRAP assays. DPPH is used in a radical scavenging assay; based on electron transfer, the antioxidant donates an electron to the DPPH radical, neutralizing and converting it to the reduced form, while the antioxidant is oxidized [60]. The DPPH radical produces an intense violet solution. When a DPPH solution is mixed with a test compound that can donate a hydrogen atom, this changes the color of the compound to colorless or light yellow as the free radicals are scavenged [61]. The FRAP assay measures the ability of the antioxidant to reduce ferric ions (Fe^3+^) to ferrous ions (Fe^2+^) [21]. An antioxidant assay assesses the ability of these phenolic and flavonoid compounds to donate an electron from a hydroxyl group to an unpaired electron of free radicals and then the antioxidant activity occurs [43,62]. The reaction of radical neutralization depends on many factors, among them the hydrogen ion concentration in the measuring system [63].

The differences in antioxidant activity found in the current systematic review and meta-analysis between *Apis mellifera* and stingless honey may be related to the presence of components such as vitamin C, vitamin E, and carotenoids, which may contribute to the higher antioxidant activity of stingless honey [8,64]. Together, these components can increase antioxidant activity and stimulate biomolecules (e.g., proteins, carbohydrates, nucleic acids, and lipids) in cells to produce reactive oxygen species, resulting in a high antioxidant response [12]. Thus, antioxidant activity results from the combined activity of a number of compounds, not just phenolic and flavonoid compounds [44]. Accordingly, Shamsudin et al. [21] found that the phenolic content, flavonoids, and antioxidant activity were higher in stingless honey than in *Apis mellifera* honey. In addition, our results showed that the *Hypotrigona* bee genera produced honey with higher antioxidant activity compared to the other stingless bee species (*p* < 0.05) (Table 2).

### 4.2. Physicochemical Composition

Our results revealed significant differences in the physicochemical parameters between stingless honey and *Apis mellifera* honey, highlighting the high moisture percentage and free acidity in contrast to the lower HMF content and diastase activity in stingless honey. In this sense, the physicochemical properties such as the color, free acidity, moisture, and HMF of honey are some of the parameters used for the authentication, the measurement of quality, and the detection of any adulteration in the product [65]. The physicochemical quality criteria of honey from the honey bee *Apis mellifera* are well specified, set by the Codex Alimentarius Commission (2001) [66], but these could not be applied for stingless bee honey. The moisture, free acidity, diastase activity, and HMF content have been shown to vary between *Apis mellifera* honey and stingless bee honey [8]. The physicochemical difference found in the current study could be related to several factors such as botanical origin, geographical area, climate, harvesting method, bee genera, and species [46]. An implication of the current study is the possibility to contribute to the determination of quality parameters that are important to know, to set quality standards for honey from stingless bees, and to prevent its adulteration [67].

#### 4.2.1. Moisture

Based on the available studies, it was found that the stingless honey had high moisture levels (>20%) to be compared with *Apis mellifera* honey; the relevance of this finding lies in the fact that moisture is a quality parameter in the set standards of honey. In addition, moisture affects viscosity, flavor, and crystallization, and is considered a useful indicator for improving storage and preservation [1,67]. The high moisture content in stingless honey is not surprising, as it has been widely reported for this type of honey [68]. This higher moisture content of stingless honey could be related to the fact that stingless bees collect nectar from the flowers of low-growing plants, which tend to produce water-rich nectar [69,70]. According to Jimenez et al. [8], the moisture content of honey from stingless bees varies according to the geographical area and the predominant vegetation. In tropical areas, polyfloral vegetation is influenced by high annual rainfall and high humidity, which may increase the water content of honey, whereas in a rainforest, honey is characterized by low water content [1,46]. Stingless bees store honey in pots made of cerumen, whereas *Apis mellifera* store honey in a hexagonal comb constructed with beeswax, which is a complex material with more than 300 identified substances [65]. During honey storage, stingless bees and honey bees use trophallaxis to reduce the water content of the collected nectar to produce honey, but the moisture content of stingless bee honey is usually higher (~31%) [71].

The high moisture content influences the characteristics of honey, as it allows microorganisms to proliferate during storage, especially yeasts, which could cause honey fermentation [7,46]. In contrast, the honey produced by *Apis mellifera* has a lower water content (<20%), which prevents the fermentation process from occurring [53]. The fermentation process could be considered as contributing to the risk of spoilage in *Apis mellifera* honey, but in stingless bee honey, it does not seem to affect the quality of the product [22]. The Codex Alimentarius establishes 20% as the maximum limit of moisture in *Apis mellifera* honey [72], so these actual standards cannot be applied to stingless bee honey due to the natural water content in each product; therefore, it is necessary to establish specific standards for stingless bee honey.

#### 4.2.2. Free Acidity

Our results showed that free acidity was higher in stingless honey compared to *Apis mellifera* honey. Free acidity is considered one of the most important parameters for assessing the freshness of honey [67]. Free acidity refers to the free natural acids present in honey, which contribute to honey flavor, stability against microorganisms, and antibacterial and antioxidant activities [73]. The high acidity could be beneficial for prolonging the shelf life of honey, as there would be no favorable conditions for the development of undesirable microorganisms [46], such as *Escherichia coli* and *Streptococcus aureus* [74,75]. Furthermore, the differences in free acidity found between honeys could be related to the floral source, the harvesting season, the storage process, and the geographical area [76,77]. The maximum limit of free acidity according to Codex Alimentarius [72] is 50 meq/kg for *Apis mellifera* honey. Stingless honey has higher levels and therefore does not comply with these regulations [57]. As mentioned above, stingless honey is not included in international honey standards. Given the potential for stingless honey to be a part of regional and international markets, it is imperative to establish parameters for this product to ensure its safe consumption, quality, and marketability.

#### 4.2.3. Hydroxymethylfurfural (HMF)

Our analysis showed lower concentrations of HMF in honey from stingless bees compared to *Apis mellifera* honey. HMF is an important parameter for freshness, overheating, and honey quality [33,78]. The differences found in HMF content can be explained by several factors such as heat, prolonged storage time, content of simple sugars, content of acids, minerals, floral sources, and environmental conditions [8,29]. Hydroxymethylfurfural is a six-carbon heterocyclic organic compound containing aldehyde and alcohol (hydroxymethyl) functional groups, formed by the degradation of sugars [70]. In relation to our results, HMF can be formed by the Maillard reaction; thus, due to the high water activity (Aw) and acidity in stingless honey, the Maillard reaction could be slow and HMF formation could be inhibited, which partially explains the reduced HMF content in stingless honey [79]. Finally, in addition to the importance of HMF content in assessing honey quality, HMF has been found to be toxic to rats and mice; potential health risks to humans associated with high concentrations cannot be excluded [80]. In this sense, the lower levels that are found in stingless honey are an advantage compared to *Apis mellifera* honey.

#### 4.2.4. Color

The high content of phenolic acids and flavonoids may explain the higher color intensity found in stingless honey compared to *Apis mellifera* honey. The importance of honey color lies in the fact that it indicates the presence of pigments, such as flavonoids and carotenoids [17]; also, the color is one of the main characteristics that consumers take into account when buying honey [81]. The color of honey varies from light yellow to dark brown; light-colored honey is usually low in mineral and phenolic compounds, while dark-colored honey is usually high in mineral and phenolic compounds [57], and the differences in honey color are associated with botanical origin, phenolic content, biochemical reactions during honey maturation, and exposure to high temperatures or light [2].

### 4.3. Limitations and Opportunity Areas

This study has certain limitations, mainly the reduced number of available studies on stingless honey and *Apis mellifera* honey that specifically address the phenolic content, antioxidant activity, and physicochemical differences between these two types of honey. Fortunately, the available data were sufficient to estimate the global effect and explore the sources of heterogeneity in the current systematic review. In addition, there is a lack of uniformity in the variables analyzed, as not all studies included the parameters relevant to this research. For this reason, some chemical characteristics could not be analyzed using a meta-analytic approach. For these variables, only descriptive statistics are presented in the supplementary materials. Finally, although the diversity of stingless bee species is considerable, the lack of studies focusing on honey produced by individual species represents a significant research gap and an area for future studies.

## 5. Conclusions

Several studies have reported a higher concentration of bioactive compounds and antioxidant activity in stingless bee honey. However, they are very heterogeneous, so it is necessary to carry out more studies on the content and profile of phenolic acids and flavonoids in stingless bee honey. The phenolic compounds are related to their antioxidant activity in vitro, and in vivo studies concerning the nutraceutical properties of honey are still scarce. It is also vital to note that the floral source, geographical area, climate, latitude, and bee species influence variances in the bioactive component content, antioxidant activity, and physicochemical composition of honey. The honey produced by stingless bees has a high level of free acidity and moisture, which does not comply with the standards laid down by the legislation and applies exclusively to honey produced by *Apis mellifera*. Therefore, it is necessary to have an exclusive quality standard for honey from stingless bees to enable better production and commercialization that will guarantee the authenticity of the products for consumers.

## Figures and Tables

**Table 1:** Standardized mean difference (SMD) and raw mean difference (RMD) of random effect model with 95% CI and a heterogeneity test of phenolic compounds, antioxidant activity, and physicochemical parameters outcomes of *Apis mellifera* and stingless bee honey.

			Effect Size				Heterogeneity		Bias
Item	Trials (*n*)	*Apis mellifera* Mean (SD) ^a^	RMD (95% CI) ^b^	*p*-Value	SMD (95% CI) ^c^	*p*-Value	*I*^2^ (%)	*p*-Value	*p*-Value ^d^
Phenols (mg GAE/100 g)	34	61.21 (28.30)	+33.69 (+7.11, +60.27)	0.01	+2.45 (−4.78, +9.70)	0.50	90.4	0.0001	0.21
Flavonoids (mg QE/100 g)	33	9.94 (8.7)	+3.59 (−0.01, +7.19)	0.05	+2.56 (−0.70, +5.83)	0.12	89.3	0.001	0.06
DPPH (μmol TE/100 g)	30	78.10 (20.21)	+37.57 (−13.60, 88.76)	0.15	+2.0 (−3.41, +7.41)	0.46	90.4	0.001	0.19
FRAP (μmol Fe(II)/100 g)	23	97.34 (7.84)	+63.39 (+15.52, +111.26)	0.009	+4.51 (+1.60, +7.42)	0.002	89.7	0.0001	0.08
Moisture (%)	31	19.54 (3.65)	+ 8.02 (+5.93, +10.11)	0.0001	+10.09 (+6.52, +13.66)	0.0001	92.6	0.0001	0.0006
HMF (mg/kg)	17	20.14 (16.27)	−11.25 (−15.88, −6.62)	0.001	−3.96 (−6.07, −1.85)	0.0002	93.5	0.0001	0.008
Free acidity (meq/kg)	19	31.32 (16.67)	+34.76 (+7.17, +62.34)	0.01	+3.17 (−0.45, +6.81)	0.08	93.6	0.0001	0.52
Color (mm/Pfund)	18	64.48 (11.93)	+10.24 (−10.22, 30.71)	0.32	+6.31 (−12.23, −0.39)	0.036	95.1	0.0001	0.101
Protein (g/kg)	22	3.49 (4.08)	−0.31 (−1.18, −0.55)	0.48	−0.83 (−3.98, +2.30)	0.60	92.6	0.0001	0.77

^a^ Mean and standard deviation of *Apis mellifera* honey. ^b^ RMD is the raw mean difference that estimates the global effect expressed in the original unit measures. ^c^ SMD is the standardized mean difference estimated of the random model. ^d^ *p*-value of Begg´s test; *p*-values > 0.05; *I*^2^ is measure of heterogeneity of random model.

**Table 2:** Meta-regression slopes and significance of covariables to outcomes with a moderate or higher heterogeneity (*I*^2^ > 25%).

	Covariates (*β*)				*R* ^2^	*I* ^2^
Item	Genera	Floral Source	Country	Latitude		
Phenols	*Melipona* −26.01 * Nannotrigona −29.43 * Partamona −27.41 *	Polyfloral −45.60 **	Cuba +69.58 *** Nigeria +63.61 *** Peru +82.04 ***	−1.19 **	87.85	59.41
Flavonoids			Cuba +18.07 *** Nigeria +19.87 *** Peru +15.31 ***	−0.46 **	9.34	97.76
FRAP	*Hypotrigona* +12.11 * Melipona −8.59 ** Plebeia −6.98 * Trigona −14.42 ***	Polyfloral +13.60 ***	Cuba −10.03 *** Nigeria −17.47 ***		83.78	74.66
Moisture	*Trigona* −14.44 *	Polyfloral +14.65 *	Cuba −15.02 ** Nigeria −16.87 ** Peru −9.89 *		53.86	91.55
HMF	*Hypotrigona* +26.55 *** Melipona +19.43 *** Plebeia +19.82 ***	Polyfloral −3.91 ***	Cuba +2.35 *		100.0	0.0
Free acidity	*Hypotrigona* +27.47 *** Melipona −7.76 *** Scaptotrigona −5.09 * Trigona −45.95 **	Polyfloral −2.53 *	Brazil +6.14 *** Cuba +2.95 * Nigeria −10.85 ***	−0.31 *	96.58	70.06
Color	*Hypotrigona* +24.48 ** Melipona +23.64 ** Plebeia +28.13 ***		Brazil −69.59 * Nigeria −30.80 *	−2.39 *	99.93	11.69

*R*^2^ = amount of heterogeneity accounted for the mixed model. Significance codes: *** < 0.001; ** < 0.01; * < 0.05.

## Data Availability

The relevant data are available from J.C.A.-H. upon reasonable request.

## Supplementary Materials

The following supporting information can be downloaded at https://www.mdpi.com/article/10.3390/antiox13121539/s1.
